# Role of consolidative thoracic radiation in extensive-stage small-cell lung cancer with first-line chemoimmunotherapy: a retrospective study from a single cancer center

**DOI:** 10.1007/s12672-023-00666-7

**Published:** 2023-05-04

**Authors:** Yuying Li, Wang Jing, Xuquan Jing, Yulan Sun, Xiaoyong Tang, Jun Guo, Yan Zhang, Hui Zhu

**Affiliations:** 1 Department of Radiation Oncology, Shandong Cancer Hospital and Institute, Shandong First Medical University and Shandong Academy of Medical Science, Jinan, 250117 Shandong Province China; 2 Shandong First Medical University and Shandong Academy of Medical Science, Jinan, 250021 Shandong Province China; 3 Department of Medical Oncology, Shandong Cancer Hospital and Institute, Shandong First Medical University and Shandong Academy of Medical Science, Jinan, 250117 Shandong Province China

**Keywords:** Small-cell lung cancer, Thoracic radiation, Immunotherapy, Toxicity, Survival

## Abstract

**Objective:**

To investigate the role of consolidative thoracic radiation (TRT) in extensive-stage small-cell lung cancer (ES-SCLC) receiving first-line chemo-immunotherapy followed by immunotherapy maintenance.

**Patients and methods:**

Outcomes of patients without disease progression after first-line chemotherapy were retrospectively reviewed (January 2020 to December 2021). Based on TRT or not, patients were allocated to TRT group or non-TRT group. Progression-free survival (PFS), overall survival (OS) and local-recurrence free survival (LRFS) were calculated by the Kaplan–Meier method and compared by log-rank test.

**Results:**

Of 100 patients, 47 received TRT and 53 non-TRT. The median follow-up was 20.3 months. The median PFS and OS in TRT were 9.1 months and 21.8 months, versus 8.8 months (*p* = 0.93) and 24.3 months (*p* = 0.63), respectively, in non-TRT. The median LRFS time in TRT was not reached, but significantly longer than 10.8 months in non-TRT (HR = 0.27, *p* < 0.01). Second-line chemotherapy significantly prolonged survival compared to that with chemo-free patients (mOS: 24.5 vs. 21.4 months, *p* = 0.026). The subgroup analysis showed a trend of patients with brain metastases benefit from TRT (21.8 versus 13.7 months, HR 0.61,* p* = 0.38) while liver metastases did not. Of 47 patients with TRT, only 10.6% of patients experienced grade 3 radiation-induced pneumonitis, while no grade 4 or 5 adverse events occurred.

**Conclusion:**

Consolidative TRT in the period of immunotherapy maintenance followed first-line chemo-immunotherapy did not prolong OS and PFS but associated with improved LRFS in ES-SCLC.

**Supplementary Information:**

The online version contains supplementary material available at 10.1007/s12672-023-00666-7.

## Introduction

Small-cell lung cancer (SCLC) is notorious due to its poor survival, while extensive-stage SCLC (ES-SCLC) exacerbated the poor prognosis further, with less than 3.0% of 5-year survival rate. In the past three decades, platinum-based chemotherapy dominated the treatment of ES-SCLC but 10 months of median survival darken the light from the 70–90% of high response rate [1, 2]. How to improve survival in ES-SCLC is an urgent issue.

Immunotherapy, generally refers to immune checkpoint inhibitors (ICIs), has broken the treatment deadlock for ES-SCLC and has brought the light to practice. IMpower133 and CASPIAN trials illustrated that ICIs added to chemotherapy significantly improved the median survival to more than 1 year [3, 4]. A growing number of studies have also shown that the addition of ICIs to chemotherapy prolonged survival [5, 6]. However, the combined strategy is lost in a bottleneck that survival is very difficult to break through, with median survival ranging from 12.3 months to 15.4 months and a 1-year survival rate of 51.9% to 60.7% [6–8]. Furthermore, worse median survival from 8.85 to 11.0 months in the real world made the combination of chemotherapy and immunotherapy even less promising [9–12]. The needs to prolong the survival of ES-SCLC were still far away to meet.

Thoracic radiation (TRT) undoubtedly benefited local control and survival of ES-SCLC in the chemotherapy era [13–15]. TRT in addition to chemotherapy improved the median survival time from 9.3 months to 17 months (*p* = 0.014) in our retrospective study [16]. Radiotherapy (RT), previously considered as a local therapy, was also an immunomodulatory factor to improve the immune microenvironment and released tumor-associated antigens. Radioimmunotherapy brings more hope, but also more mysteries, for instance, the toxicity of radioimmunotherapy, the window of RT to ICIs, and the fractionation of RT. Regardless of the toxicity of TRT plus immunotherapy reported was controllable [17], the data from the real world was still lacking, however. What is unknown is whether TRT could further enhance the benefit of ICIs maintenance on the outcomes of ES-SCLC.

We sought to evaluate progression-free survival (PFS) and OS outcomes for ES-SCLC treated with first-line chemoimmunotherapy followed by ICIs maintenance, in the context of TRT or not in the period of maintained ICIs.

## Materials and methods

### Patients

Between January 2020 and December 2021, patients with treatment-naïve ES-SCLC diagnosed by histopathology or cytology were retrospectively collected at Shandong cancer hospital and institute. The medical records of patients should contain whole-body systemic evaluation before treatment, including cervical (ultrasound examination was also eligible), chest and abdomen contrast-enhanced computed tomography (CT), brain contrast-enhanced MRI or CT, or positron emission tomography (PET)-CT (not routinely used in our cancer center). All patients were treated with chemotherapy concomitant with ICIs followed by ICIs maintenance (with or without thoracic radiation). Patients with disease progression after 4 cycles of chemo completed according to the criteria of RECIST version 5.0 were excluded. Considering TRT given or not, patients were divided into TRT group and non-TRT group.

All procedures performed in studies involving human participants were in accordance with the ethical standards of Shandong Cancer Hospital and with the 1964 Helsinki declaration and its later amendments or comparable ethical standards. This study was approved by the appropriate institutional review board, and the requirement for informed consent was waived.

### Treatment approach

Platinum-based chemotherapy was used in this study. Thoracic radiation was performed using intensity modulation radiation therapy technique with 6MV photon therapy. The gross target volume included the residual thoracic disease and positive lymph nodes, and the clinical target volume included gross target volume + 8 mm margin and nodal regions involved before. Concomitant immunotherapy usually started on the first day of every treatment cycle and before chemo-agents. Immunotherapy maintenance was conducted every 21 days until disease progression or intolerable toxicity.

### Statistical analyses

Demographics and clinical characteristics were compared between patients given TRT and non-TRT. Two-sample *t*-tests or Wilcoxon ranked sum tests were used to evaluate the difference of continuous variables and chi-square or Fisher’s exact tests for categorical variables between the two groups. Adverse events were evaluated according to the criteria of CTCAE v5.0. PFS (PFS2) was measured from the date of first-line (second-line) systematic therapy given to disease progression or death from any cause, or to the date of censor. OS was measured from the first day of chemotherapy to the date of death or last follow-up. Locoregional recurrence-free survival (LRFS) was defined as the duration between the date of first-line chemotherapy to the date of locoregional recurrence or death, whichever occurs first [18]. Locoregional disease refers to local tumor disease and local–regional lymph nodes generally, specifically lesions within the radiation field in this study. The Kaplan–Meier method was performed to evaluate PFS, OS and LRFS for the two groups, and comparisons were made with the log-rank test. *P* < 0.05 was considered to be statistical significance. Statistical analyses were conducted with SPSS 23.0 (SPSS, IBM Corp., Armonk, NY).

## Results

### Patients

**Fig. 1:**
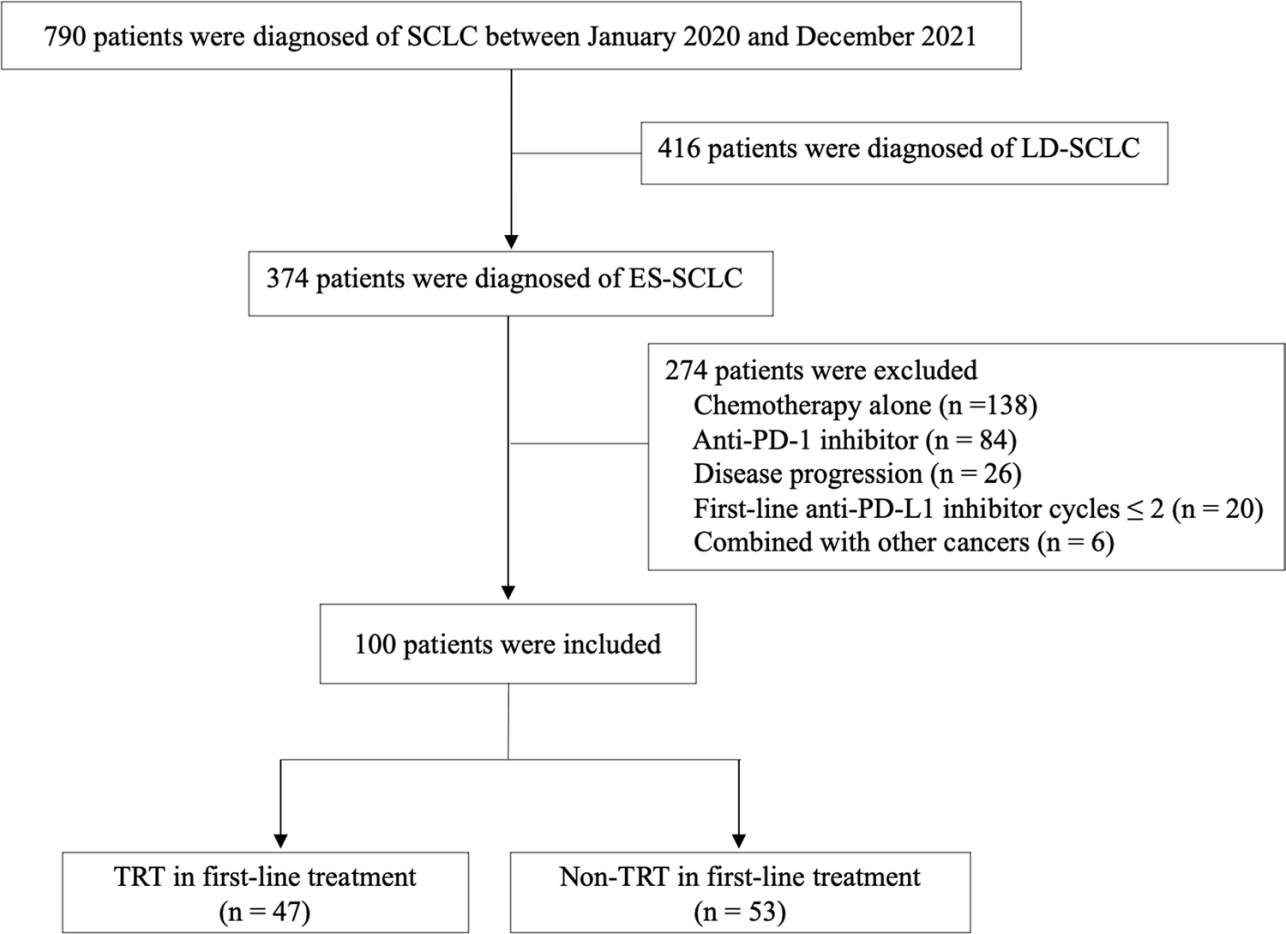
Flow diagram of the study participants

A total of 790 patients were screened as having been treated from January 2020 to December 2021 in our cancer center. As indicated in Fig. 1, 416 patients were excluded for limited-stage SCLC. Then, 274 patients were excluded due to chemotherapy alone (n = 138), PD-1 inhibitor used (n = 84), disease progression (n = 26), anti-PD-L1 ≤ 2 cycles in first-line treatment (n = 20), and concomitant with another malignancy (n = 6). Eventually, 100 patients were analyzed in our study, whereas 47 (47.0%) in the TRT group and 53 (53.0%) in the non-TRT group. The clinical characteristics of patients in TRT or non-TRT were well-balanced (Table 1). For the whole cohort, most of patients were male (84.0%), < 65 years (66.0%), KPS ≥ 80 (90.0%), smoking history (63.0%), no diabetes (91.0%), and N2 or N3 stage (93.0%). The median age of whole group is 60 years (interquartile range [IQR): 55–66). Only 4.0% of patients had underlying lung disease at baseline, 2 tuberculosis, 1 COPD, and 1 allergic asthma. Furthermore, 34.0%, 25.0%, and 30.0% of patients were with brain metastases, bone metastases and liver metastases at baseline, separately. ICIs included Durvalumab 1500 mg and Atezolizumab 1200 mg, Q3 weeks each. Durvalumab (59.0%) is the most frequently used anti-PD-L1 checkpoint inhibitor, followed by Atezolizumab (41.0%). Before TRT administration, the response of extrathoracic disease was complete response (19.1%, 9/47), partial response (61.7%, 29/47), and stable disease (19.1%, 9/47).

**Table 1:** Clinical features of ES-SCLC treated with TRT or not

	TRT group (n = 47, %)	Non-TRT group (n = 53, %)	*p* value
Age (years)			
Median (IQR)	59 (52–65)	63 (56–67)	
< 65	34 (72.3)	32 (60.4)	0.21
≥ 65	13 (27.7)	21 (39.6)	
Gender			
Male	37 (78.7)	47 (88.7)	0.17
Female	10 (21.3)	6 (11.3)	
KPS			
≥ 80	43 (91.5)	47 (88.7)	0.64
< 80	4 (8.5)	6 (11.3)	
Smoking history			
Never	17 (36.2)	20 (37.7)	0.87
Previous or current	30 (63.8)	33 (62.3)	
Diabetes			
Yes	3 (6.4%)	6 (11.3)	0.39
No	44 (93.6)	47 (88.7)	
Brain metastases			
Yes	17 (36.2)	17 (32.1)	0.67
No	30 (63.8)	36 (67.9)	
Liver metastases			
Yes	10 (21.3)	20 (37.7)	0.07
No	37 (78.7)	33 (62.3)	
Bone metastases			
Yes	12 (25.5)	13 (24.5)	0.91
No	35 (74.5)	40 (75.5)	

ES-SCLC: extensive-stage small-cell lung cancer; TRT: thoracic radiation; IQR: interquartile rage; KPS: Karnofsky performance scale

All patients received at least 4 cycles of chemotherapy with Cisplatin/carboplatin and etoposide. Cisplatin (75 mg/m^2^, day 1) or carboplatin (AUC 5 or 6, day 1) were given intravenously every 3 weeks up to 6 cycles as well as etoposide delivered with 100 mg/m^2^ on day 1–3 or 100 mg on day 1 to day 5. The median cycles of chemotherapy were 6 in the TRT group as well as non-TRT group (IQR: 5–6, each). Generally, the prescriptions of 60 Gy/30fx and 50 Gy/25fx with 2.0 Gy per fraction, and 45 Gy/15fx and 30 Gy/10fx with 3 Gy per fraction were commonly used in our cancer center, which contains 19.1% (9/47), 36.2% (17/47), 29.8% (14/47) and 14.9% (7/47) of patients correspondingly. Among 47 patients receiving TRT, the median dose of TRT is 50 Gy (IQR: 45–54). The median cycle of anti-PD-L1 therapy in the first-line for the whole cohort is 7 cycles (IQR 6–9), while 7 cycles (IQR: 6–9) in TRT as well as in non-TRT (IQR: 6–10). The median interval time from chemotherapy completion to TRT was 31 days (IQR: 12–44.5). Ten (21.3%) patients terminated ICIs in the period of TRT. Only 3 patients received prophylactic cranial irradiation (1 in TRT, 2 in non-TRT). All 35 patients with brain metastases were treated with cranial irradiation.

Second-line systematic therapy was delivered in 88.4% (61/69) of patients, whereas the remaining 8 patients were not given sequent therapy due to poor performance status (n = 5) and refused further treatment (n = 3). Second-line therapy consists of chemotherapy, immunotherapy and antiangiogenetic therapy (Table 2). The frequent second-line therapy was chemotherapy + immunotherapy (37.7%, 23/61), followed by chemotherapy alone (19.7%, 12/61) and chemotherapy + anti-angiogenetic therapy (13.1%, 8/61). ICIs continued in 62.3% (38/61) of patients in second-line were based on the following 2 reasons: ICIs used free supported by Cancer Assistance Program of Red Cross Society of China, and benefits from continued ICIs judged by physicians. In addition, 2 cases switched to PD-1 agents after the progression of PD-L1.

**Table 2:** Treatment details in the present study

	TRT group (n = 47, %)	Non-TRT group (n = 53, %)	*p* value
First-line anti-PD-L1 cycles			
Median (range)	7 (2–26)	7 (2–23)	
≥ 6	36 (76.6)	43 (81.1)	0.58
< 6	11 (23.4)	10 (18.9)	
First-line anti-PD-L1 agents			
Durvalumab	28 (59.6)	31 (58.5)	0.91
Atezolizumab	19 (40.4)	22 (41.5)	
TRT dose			
≥ 45	40 (85.1)	–	
< 45	7 (14.9)	–	
PCI			
Yes	1 (2.1)	2 (3.8)	0.39
No	46 (97.9)	51 (96.2)	
PD after first-line treatment			
Yes	39 (83.0)	39 (73.6)	0.26
No	8 (17.0)	14 (26.4)	
Second-line treatment after PD			
Yes	31 (79.5)	30 (76.9)	0.78
Chemo* + immunotherapy	11 (35.5)	12 (40.0)	
Chemo + anti-angiogenesis^#^	4 (12.9)	4 (13.3)	
Immunotherapy + anti-angiogenesis	1 (3.2)	3 (10.0)	
Others	15 (48.4)	11 (36.7)	
No	8 (20.5)	9 (23.1)	

TRT: thoracic radiation; PCI: prophylactic cranial irradiation; PD: progressive disease

*Chemotherapy: platinum, taxanes, irinotecan, temozolomide and others. Immunotherapy: durvalumab, atezolizumab, and others

^#^Anti-angiogenesis: anlotinib

### Patterns and treatment of disease progression

After the first-line therapy, 69 patients experienced disease progression eventually, including 35 in TRT and 34 in non-TRT. The rate of intrathoracic progression was 20.0% (7/35) in the TRT group, versus 55.9% (19/34) in the non-TRT group (*p* = 0.003). Of 19 patients with progressive intrathoracic disease in non-TRT, 6 patients undertook salvage thoracic radiation. The rate of extrathoracic progression in TRT was significantly worse than that in non-TRT (91.4% [32/35] vs. 67.6% [23/34], *p* = 0.02). Among 32 patients with distant disease progression in the TRT group, 23 patients experienced new brain metastases (n = 17) or brain lesions progression (n = 6), while it was 16 (8 new lesions) of 23 patients with brain malignancy in the non-TRT group.

### Survival

#### Evaluating the survival benefit of TRT

**Fig. 2:**
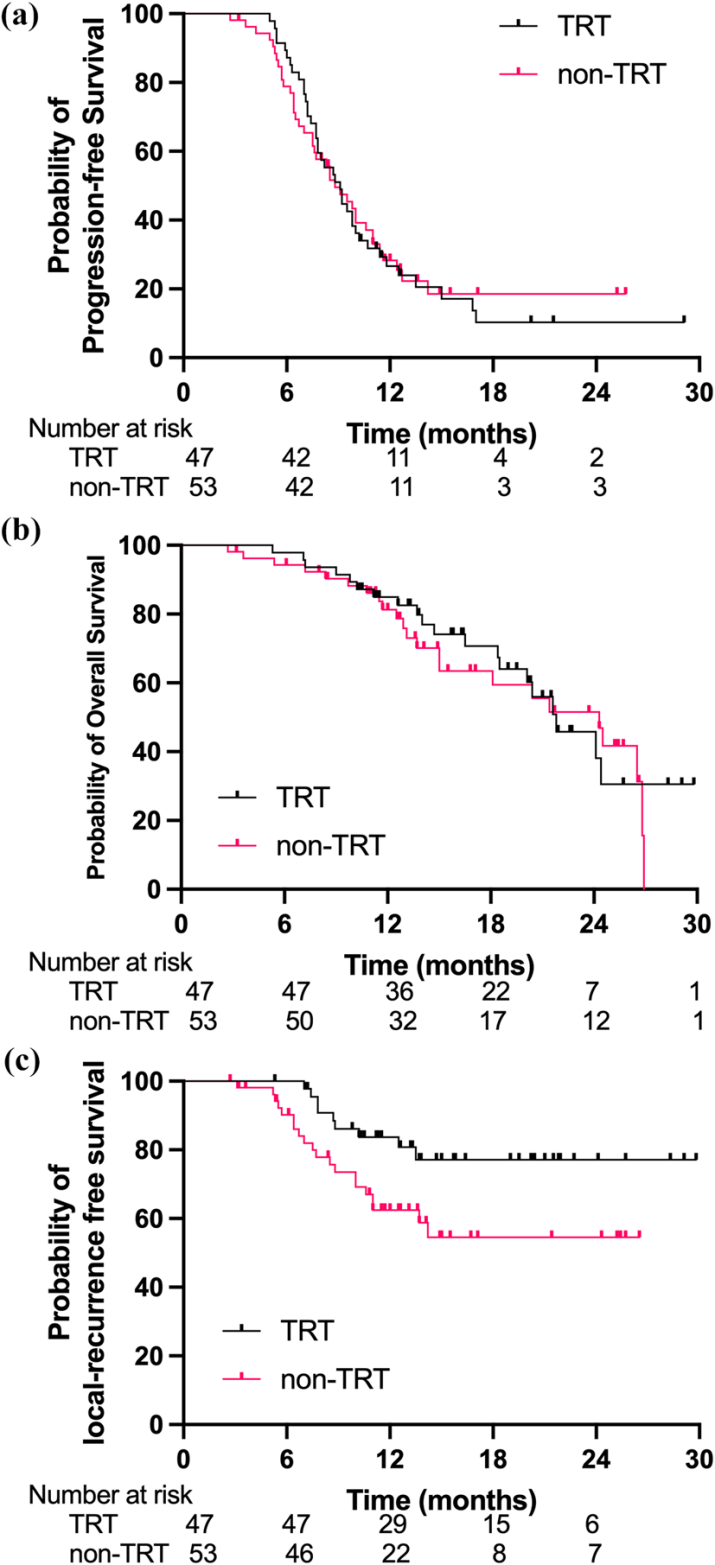
Progression-free survival (PFS), overall survival (OS) and local-recurrence free survival (LRFS) of patients with TRT or not. **a** PFS; **b** OS; **c** LRFS

The median duration of follow-up for the cohort was 20.3 months (IQR: 12.7–25.4). To the time of the data lock on November 01, 2022, 43 patients (43.0%) had died, 20 (42.5%, 20/47) in the TRT group and 23 (43.4%, 23/53) in the non-TRT group. The median PFS and OS for the whole group were 9.1 months and 21.8 months, respectively (Supplementary Fig. 1). For patients with TRT, the median PFS and OS were 9.1 months and 21.8 months, versus 8.8 months (*p* = 0.93) and 24.3 months (HR = 0.90, 95% CI 0.49–1.63, *p* = 0.63), respectively, in non-TRT (Fig. 2a, b). OS rates at 12-months and 18-months were 85.0% and 70.7% in the TRT group versus 81.3% and 63.5%, respectively, in the non-TRT group. The median LRFS time is not reached in TRT, which was significantly longer than 10.8 months in non-TRT (HR = 0.27, 95% CI 0.13–0.53, *p* < 0.01; Fig. 2c).

We performed a subgroup analysis stratified by liver and brain metastases. For patients with liver metastases, the median OS time was 13.3 months in TRT versus 15.0 months in non-TRT (HR 1.80, 95% CI 0.66–4.95, *p* = 0.21; Supplementary Fig. 2a), whereas it was 24.1 months and 24.5 months, respectively, for patients without liver metastases (HR 0.69, 95% CI 0.31 − 1.51, *p* = 0.34; Supplementary Fig. 2b). Among patients with brain metastases, the addition of TRT prolonged median OS time (21.8 versus 13.7 months) but without significant difference (HR 0.61, 95% CI 0.19–1.96, *p* = 0.38; Supplementary Fig. 3a); however, this was reversed for patients without brain metastases (18.5 months TRT versus 24.3 months non-TRT, *p* = 0.69; Supplementary Fig. 3b).

#### Univariate and multivariate analyses on overall survival

To further explore which factors mostly contribute to survival, univariate and multivariate analyses were conducted. Univariate analysis showed that KPS ≥ 80 and liver metastases correlated with OS (Table 3). These associations with N stage and bone metastases held in multivariate analyses, that is, liver metastases (OR 2.58, 95% CI 1.33–4.98, *p* = 0.01) and N stage ([N0-1] OR 0.41, 95% CI 0.20–0.82, *p* = 0.01) independently predicted OS.

**Table 3:** Univariate and multivariate analyses of factors influencing overall survival among all patients

Variables	Univariate analysis			Multivariate analysis		
	OR	95% CI	*p*	OR	95% CI	*p*
Gender (male vs female)	0.54	0.22–1.29	0.16			
KPS score (≥ 80 vs < 80)	0.37	0.15–0.89	0.03	0.39	0.15–1.06	0.07
Smoking history (yes vs no)	1.26	0.68–2.35	0.46			
T stage (≤ 2 vs ≥ 3)	0.80	0.44–1.47	0.48			
N stage (≤ 1 vs ≥ 2)	0.55	0.29–1.04	0.07	0.41	0.20–0.82	0.01
Brain metastases (yes vs no)	1.03	0.53–2.02	0.92			
Liver metastases (yes vs no)	2.32	1.25–4.29	0.01	2.58	1.33–4.98	0.01
Bone metastases (yes vs no)	1.80	0.93–3.46	0.08	1.18	0.57–2.44	0.66
First-line chemo cycles (≥ 6 vs < 6)	1.33	0.65–2.73	0.43			
First-line anti-PD-L1 cycles (≥ 6 vs < 6)	0.63	0.75–3.37	0.22			
Thoracic RT (yes vs no)	0.63	0.47–1.68	0.63			
Multiple distant sites (≤ 5 vs > 5)	0.67	0.33–1.37	0.28			

#### Impact of second-line therapy on survival

Considering potential contributions of second-line therapy to overall survival, we performed further analysis only in patients receiving second-line therapy. The median PFS2 in TRT was slightly longer than that in non-TRT but without significant difference (7.8 vs. 7.0 months, *p* = 0.35; Supplementary Fig. 4). Stratification based on the main treatment modes, survival of patients with second-line chemotherapy was significantly longer than that with chemo-free patients (mOS: 24.5 vs. 21.4 months, *p* = 0.026; Supplementary Fig. 5). However, the survival of patients with immunotherapy or anti-angiogenetic therapy was no statistical difference compared to those who did not receive corresponding treatment (not detailed).

#### Treatment adverse events

The grade and incidences of radiation-induced pneumonitis (RIP) are listed in Supplementary Table 1. The incidence of grade ≥ 2 RIP was 29.8%, while only 10.6% of patients underwent grade 3 RIP, and none of patients experienced grade 4 or worse RIP. In addition, grade ≥ 3 of hematological toxicity was worse than that in the non-TRT group (44.7% TRT versus 26.4% non-TRT,* p* = 0.04; Supplementary Table 2). No grade 5 hematological toxicity occurred. The most frequent hematological toxicity was neutropenia (36.2%), followed by thrombocytopenia (6.4%), in the TRT group, compared to 24.5% and 1.9%, respectively, in the non-TRT group. In addition, no grade ≥ 3 treatment-related cardiac events were observed in the present study.

## Discussion

Our study indicated that TRT was correlated with improved LRFS compared to that receiving chemo-immunotherapy only but failed to prolong the PFS and OS. Further subgroup analyses indicated that TRT had a trend of survival benefits in patients with brain metastases. In addition, TRT-induced RIP was also acceptable while no grade ≥ 4 pulmonary toxicities occurred. Regardless of no benefits of TRT on survival, TRT was still a potentially potent strategy for ES-SCLC due to the possibility of remarkable LRFS translating into survival benefits in selected settings.

The magnitude of survival benefit seen with consolidative TRT in the period of ICIs maintenance was not significant compared to that maintained ICIs without TRT in ES-SCLC, suggesting that the administration of TRT as consolidative therapy needs to be further investigated in certain subgroups. TRT benefits survival from a phase III randomized study in the era of two-dimensional radiotherapy ignited the study of TRT in ES-SCLC; however, TRT-mediated local control did not show an advantage [19]. CREST study indicated hypofractionated TRT in ES-SCLC patients responded first-line chemotherapy can benefit 2-year OS (13% vs. 3%, *p* = 0.004) and local control (19.8% vs. 46.0%) compared to that without TRT [13]. Based on the potent efficacy of TRT for ES-SCLC in the chemotherapy era, it seems to be more pivotal to investigate the role of TRT in ES-SCLC in the immunotherapy era. However, no survival benefits derived from TRT were observed but TRT-mediated LRFS was significantly prolonged in our study. That may be due to the survival benefit from TRT being weakened by the long ICI-induced survival; in addition, the second-line regents may contribute to the prolonged survival. Third, more proportion of distant progression may attenuate the benefits of TRT derived.

However, the subgroup that can benefit from TRT were still hard to identify yet. Specific metastatic organ or metastatic load seems to have an impact on survival. Fewer metastases or oligometastasis seem to contribute to better survival but with liver metastases were not [20, 21]. Advantage of maintained immunotherapy on survival does not seem to be true for cases with liver metastases. Liver metastases (OR 5.69, *p* = 0.069) were a trend with prognostic factors associated with reaching the maintenance phase in IMpower133 exploratory analysis [22]. In our study, liver metastases also decreased survival regardless of TRT delivered or not compared to those without liver metastases (mOS: 15.0 vs. 24.4 months, *p* = 0.004) and was a negatively independent factor on OS (OR 2.58, *p* = 0.01).

We noticed that patients with brain metastases have a trend to benefit from TRT. Unlike liver metastases without local-RT, all patients with brain metastases were treated with cranial radiation concomitantly with immunotherapy in 94.1% (32/34) of patients. The trend of better outcomes in BM with TRT may be attributed to the treatment of local lesions; moreover, broken blood–brain barrier due to brain radiation further promotes the permeability of immune agents. Previous studies demonstrated that radioimmunotherapy-induced abscopal effect was rare [23], while multi-site radiation with high- and low-dose for selected lesions might be a curative strategy for systemic disease control [24]. In particular, the tumor immunogenicity (hot or cold), metastatic sites and numbers should be considered cautiously in the context of numerous unsolved mysteries of radioimmunotherapy; moreover, one-size regimen could not solve personalized problems [25]. Radiation as an immunomodulatory drug reverses tumor immune desertification relying upon mobilizing both adaptive and innate immunity [26]. Therefore, only TRT may be insufficient for systemic disease control, particularly in ES-SCLC. Liver radiation plus immunotherapy promoted systemic antitumor immunity, and was relevant to prolonged survival in practice [24, 27–29]. Therefore, local treatment of extrathoracic residuals integrated with TRT may be a potentially crucial approach to improve the survival of ES-SCLC.

Several factors affect the survival of TRT to ES-SCLC, not just those mentioned above. The patterns of radiation, such as radiation dose, hyper- or hypo-fractionation, RT frequency, may affect local control and survival [30–32]. In addition, the time to TRT given was more confusing, while TRT administration after 4–6 cycles of chemotherapy like this study or until thoracic disease progressed or other times were still unclear. Furthermore, second-line treatment is very important in ES-SCLC, but lacks the optimal agents after first-line therapy failed. In the present study, second-line chemotherapy significantly prolonged OS compared to chemo-free strategy in 61 patients with disease progression (mOS: 24.5 vs. 21.4 months, *p* = 0.026). However, second-line therapy based on immunotherapy or antiangiogenesis did not make a significance on survival. It should be noted that this conclusion cannot be generalized to a larger population because of the results concluded from a subset of a small sample.

Based on concerns about the increased toxicity of radiotherapy combined with ICIs, ICIs were commonly suspended during TRT in clinical practice. However, the toxicity of concurrent TRT and pembrolizumab in limited-stage SCLC was acceptable [33]. Moreover, TRT with pembrolizumab concurrently in ES-SCLC was also well-tolerated, with only 6% of patients experiencing grade 3 adverse events and no grade 4–5 toxicities observed [17]. Another retrospective study also showed that only 15% of patients occurred pneumonitis (3 grade 2 and 3 each) in patients with concurrent atezolizumab and TRT [34]. In our study, of 37 patients received TRT and ICIs simultaneously, only 10.6|% of patients underwent grade 3 RIP, while none experienced more serious adverse events.

This study has its own merits. First, this study thoroughly analyzed the impact of TRT versus non-TRT and second-line treatment on survival in ES-SCLC receiving chemo-immune agents, as well as on local control and toxicity, verified the superiority of TRT on local control and confirmed the feasibility of the combination of radiotherapy and immunotherapy. Second, this study creatively proposed that the management of distant lesions by local therapy might be a potentially curative approach for ES-SCLC. The attitude to metastatic sites should be more aggressive in the subsettings. However, small sample sizes of this retrospective study from a single cancer center increased the inherent flaw of selection bias, which further contribute to the insufficient power of statistical efficacy in certain subgroup analyses. In addition, the correlation of radiation parameters and survival was not performed, such as radiation dose, RT technique, etc. Ultimately, the impact of metastatic load on survival was not further explored, such as numbers of metastatic lesions and/or organs.

In conclusion, the consolidative TRT in the period of ICIs maintenance did not prolong survival, but increased LRFS, and the trend of TRT benefits in subgroups made it more worthy to study in the era of radioimmunotherapy. The results of TRT on outcomes in ES-SCLC need to be validated in a prospective clinical trial.

## Supplementary Information

Additional file1

## Data Availability

All data included in this study are available upon request by contact with the corresponding author.
