# Supplementary material for: Role of consolidative thoracic radiation in extensive-stage small-cell lung cancer with first-line chemoimmunotherapy: a retrospective study from a single cancer center
Source: Discov Oncol. 2023 May 4;14:55. doi: 10.1007/s12672-023-00666-7 (PMC10160328; doi:10.1007/s12672-023-00666-7)
Supplement: Supplementary file 1 — Additional file1 [file 12672_2023_666_MOESM1_ESM.zip › Supplementary Materials for Review/Supplementary Tables.docx]

Supplementary Tables

Table 1. Radiation pneumonitis.

| Grade | TRT group (n = 47) |
| --- | --- |
| Grade 0 | 11 (23.4%) |
| Grade 1 | 22 (46.8%) |
| Grade 2 | 9 (19.1%) |
| Grade 3 | 5 (10.6%) |
| Grade 4 - 5 | 0 |

Table 2. Adverse events of hematological toxicity (Grade ≥ 3).

|  | TRT group  (n = 47) | Non-TRT group  (n = 53) |
| --- | --- | --- |
| Neutropenia | 17 (36.2%) | 13 (24.5%) |
| Anemia | 1 (2.1%) | 0 |
| Thrombocytopenia | 3 (6.4%) | 1 (1.9%) |
