# Supplementary figures and images for: Role of consolidative thoracic radiation in extensive-stage small-cell lung cancer with first-line chemoimmunotherapy: a retrospective study from a single cancer center
Source: Discov Oncol. 2023 May 4;14:55. doi: 10.1007/s12672-023-00666-7 (PMC10160328; doi:10.1007/s12672-023-00666-7)

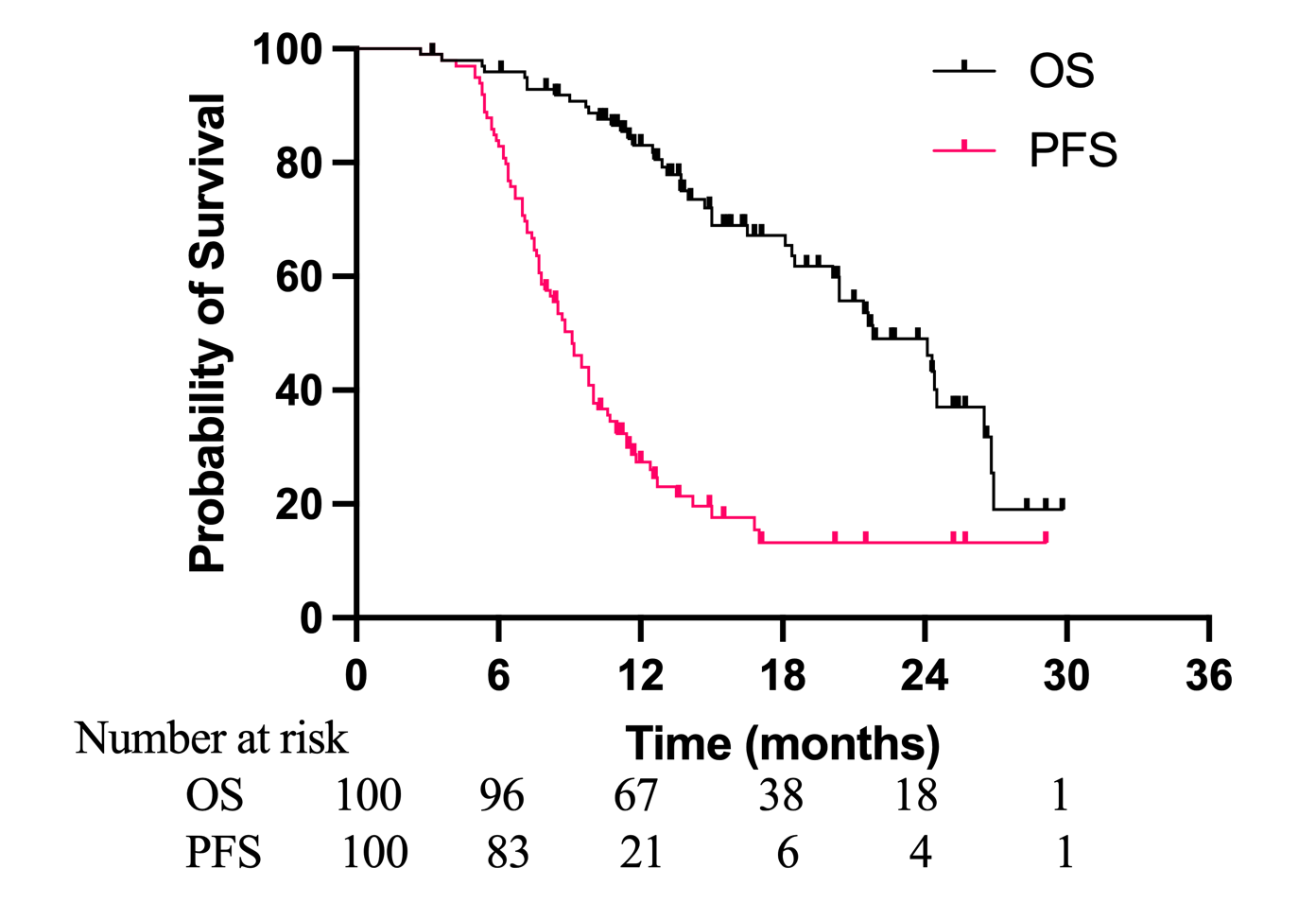

Supplement: Supplementary file 1 — Additional file1 [file 12672_2023_666_MOESM1_ESM.zip › Supplementary Materials for Review/Supplementary Figure 1.tiff]

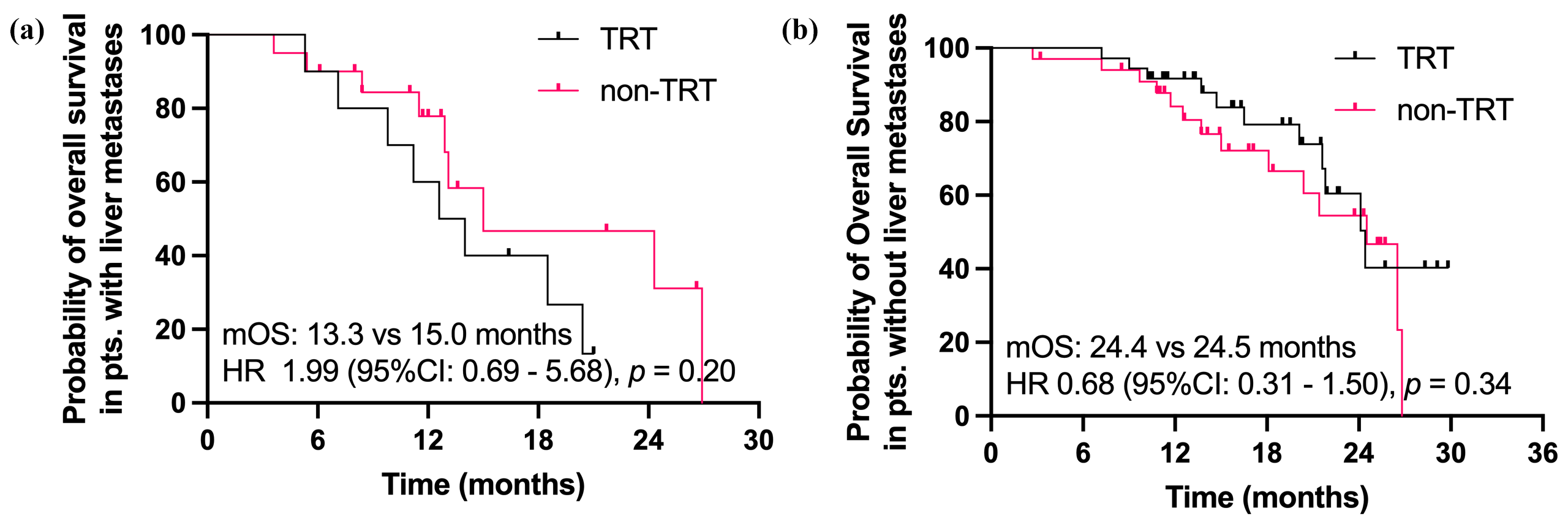

Supplement: Supplementary file 1 — Additional file1 [file 12672_2023_666_MOESM1_ESM.zip › Supplementary Materials for Review/Supplementary Figure 2.tiff]

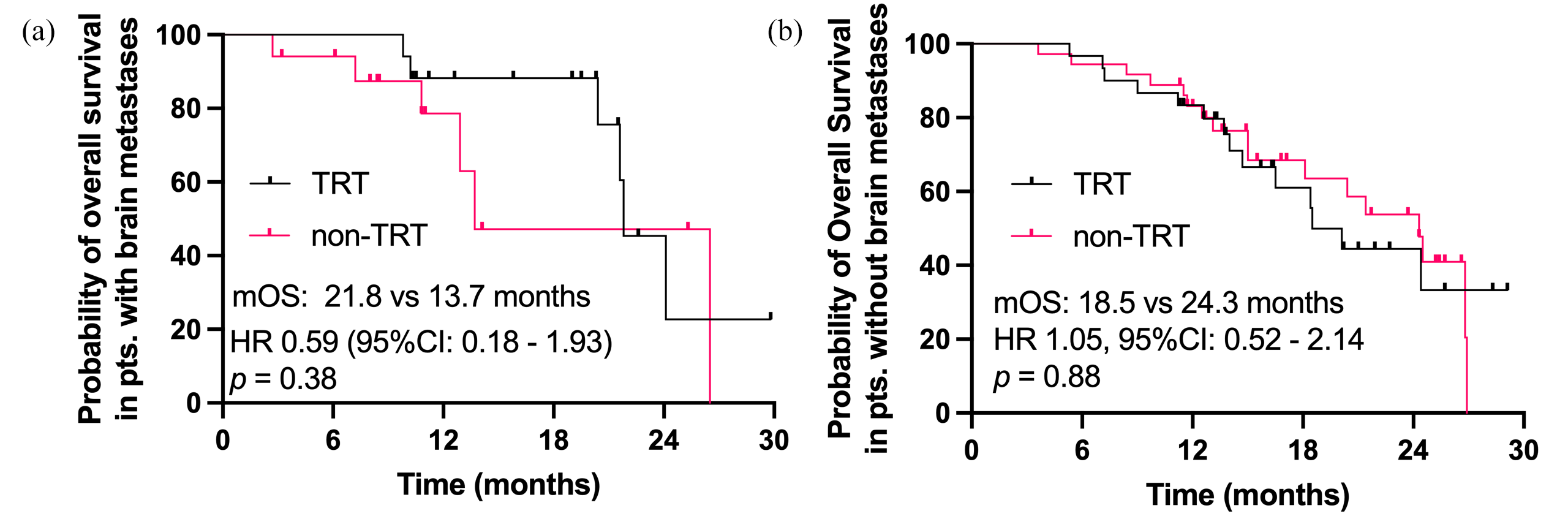

Supplement: Supplementary file 1 — Additional file1 [file 12672_2023_666_MOESM1_ESM.zip › Supplementary Materials for Review/Supplementary Figure 3.tif]

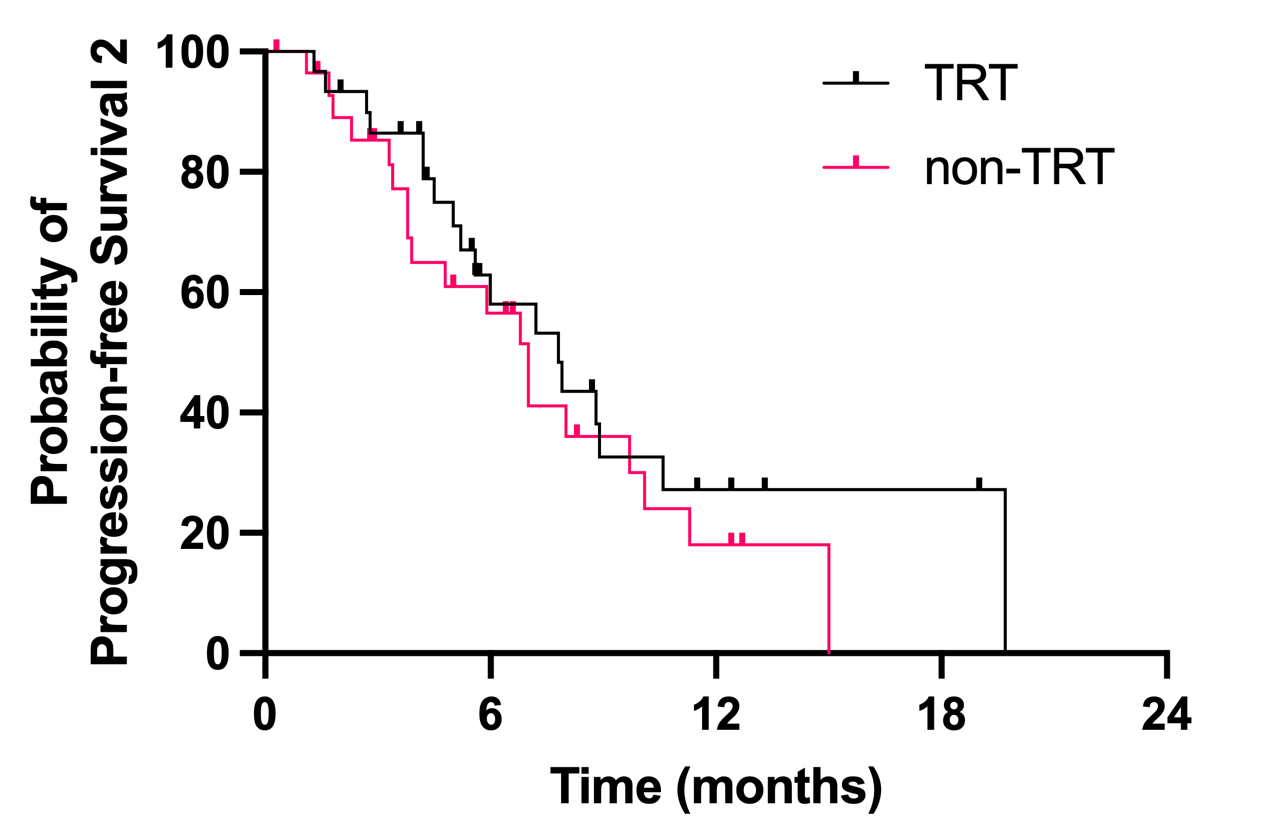

Supplement: Supplementary file 1 — Additional file1 [file 12672_2023_666_MOESM1_ESM.zip › Supplementary Materials for Review/Supplementary Figure 4.tiff]

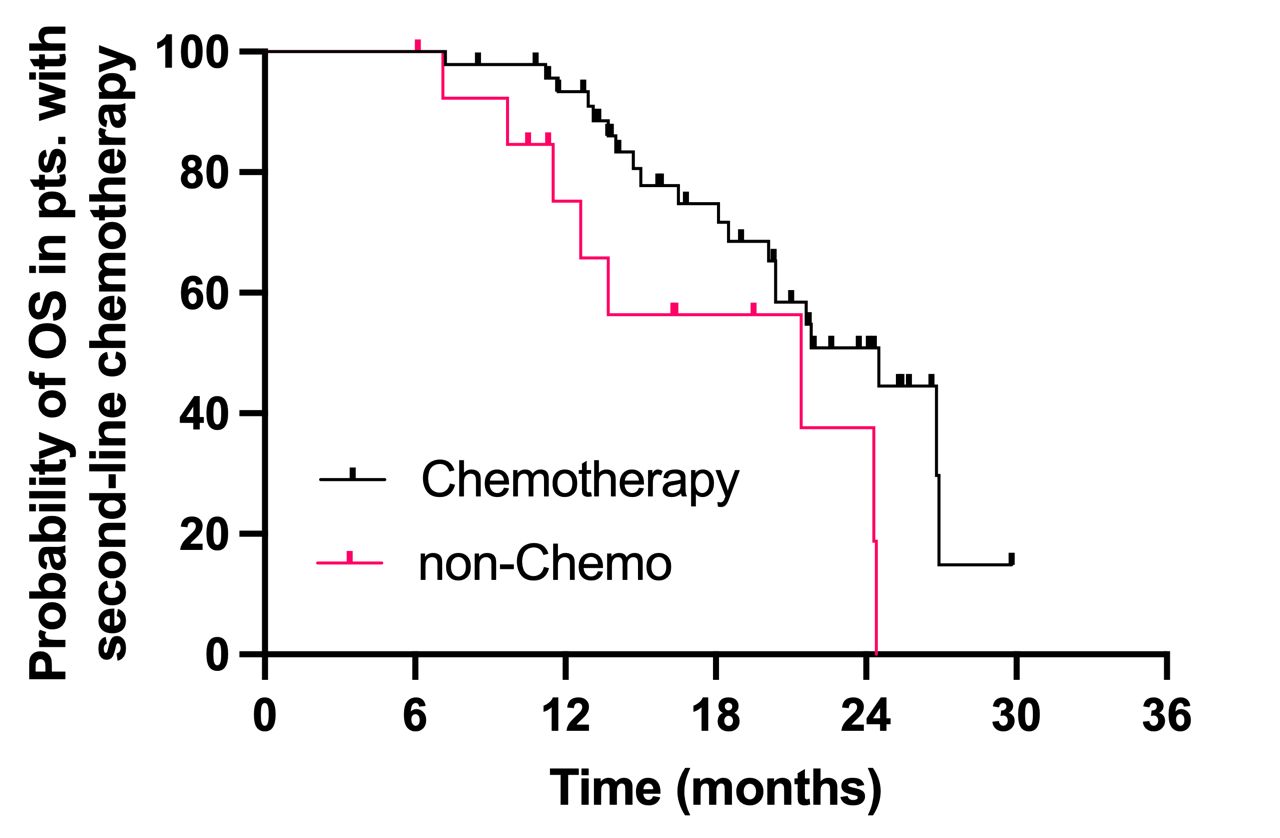

Supplement: Supplementary file 1 — Additional file1 [file 12672_2023_666_MOESM1_ESM.zip › Supplementary Materials for Review/Supplementary Figure 5.tiff]
